# RISK STRATIFICATION PROTOCOL FOR PERFORMING TOTAL KNEE ARTHROPLASTY

**DOI:** 10.1590/1413-785220253303e287197

**Published:** 2025-08-18

**Authors:** Fabrício Bolpato Loures, Guilherme de Mattos Queiroz, Danielle Lopes Rosa, Guilherme Morgado Runco, Liszt Palmeira de Oliveira, Vinícius Schott Gameiro

**Affiliations:** 1 Universidade Estadual do Rio de Janeiro (UERJ), Hospital Universitário Pedro Ernesto, Rio de Janeiro, RJ, Brazil.; 2 Universidade Federal Fluminense, Faculdade de Medicina, Niteroi, RJ, Brazil.

**Keywords:** Arthroplasty, Risk Factors, Knee, Risk Assessment, Blood, Intensive Care Units, Artroplastia, Fatores de Risco, Joelho, Medição de Risco, Sangue, Unidade de Terapia Intensiva

## Abstract

**Objective:**

the main objective of the study was to evaluate a screening protocol for performing total knee arthroplasty (TKA) without postoperative admission to an intensive care unit (ICU). The secondary objective was to evaluate the postoperative blood transfusion rate.

**Method:**

between January 2020 and December 2021, 270 TKA were performed, following a clinical protocol: age up to 75 years, body mass index up to 35 kg/m2, ASA score classification I or II, non-smoker, without history of ischemic disease (coronary or cerebral), creatinine clearance greater than 60 mL/min, hemoglobin greater than 12 g/dL and osteoarthritis with deformity treatable with primary prosthesis (fixed or rotating base). The need for conversion to ICU and the blood transfusion rate were assessed.

**Results:**

270 patients underwent surgery during the study period and only one required admission to the ICU, giving the screening protocol a positive predictive value of 99.6%. There was no indication for blood transfusion in any case in the sample.

**Conclusion:**

The proposed screening protocol proved to be effective, allowing total knee arthroplasty to be performed without the prior need for ICU bed reservation, blood typing and/or blood products reservation, without compromising patient safety. ***Level of Evidence IV; Case Series***.

## RESUMO

**Objetivo:**

o objetivo principal do estudo foi avaliar um protocolo de triagem para realização de artroplastia total do joelho (ATJ) sem admissão pós-operatória em unidade de terapia intensiva (UTI). O objetivo secundário foi avaliar o índice de transfusão sanguínea pós-operatória.

**Método:**

entre janeiro de 2020 e dezembro de 2021, foram realizadas 270 ATJ, seguindo um protocolo clínico, sendo: idade até 75 anos, índice de massa corporal até 35 kg/m^2^, classificação do score ASA I ou II, não tabagista, sem história de doença isquêmica (coronariana ou cerebral), clearance da creatinina maior que 60 mL/min, hemoglobina maior que 12 g/dL e osteoartrite com deformidade tratável com prótese primária (base fixa ou rotatória). Foram avaliados a necessidade de conversão para UTI e o índice de hemotransfusão.

**Resultados:**

270 pacientes foram operados no período do estudo e apenas um apresentou necessidade de internação em UTI, conferindo ao protocolo de triagem um valor preditivo positivo de 99,6%. Não houve indicação de hemotransfusão em nenhum caso da amostra.

**Conclusão:**

O protocolo de triagem proposto demonstrou ser eficaz, permitindo a realização de ATJ sem a necessidade prévia de reserva de leito em UTI, sem a tipagem sanguínea e/ou reserva de hemoderivados, não comprometendo a segurança do paciente. ***Nível de Evidência IV; Série De Casos***.

## INTRODUCTION

Osteoarthritis (OA) is the leading cause of musculoskeletal disability in the elderly population. It is a major global public health problem, being even more prevalent in developed countries than in developing countries.^1^ It is estimated that in Brazil, 33% of the population over the age of 25 has some form of osteoarthritis, corresponding to 39 million people.^2^ The incidence of the disease is expected to increase significantly in the coming years, driven by an aging population and rising obesity rates.^3^ OA has high treatment costs and also a high social cost, having been the second leading cause of sickness benefits granted by the National Social Security Institute (INSS) in 2019.^4^ Due to anatomical and biomechanical factors, the knee is a commonly affected joint, accounting for about 85% of healthcare spending on the disease and representing a substantial and growing share of healthcare resources.^5^

Total knee replacement (TKR) is the recommended procedure for severe cases of osteoarthritis that are resistant to conservative treatment. It can effectively relieve pain, restore function, and allow early return to daily activities, making it one of the most successful surgical procedures of the century.^6^ In the United States, TKR is the most common surgery in hospitalized patients, with 700,000 TKRs performed each year.^7^ Projections indicate that this number is expected to reach 1.3 million by 2030.^8^ In Brazil, approximately 13,000 TKRs were performed in 2019.^9^ However, the national average of 6.29 TKRs per 100,000 inhabitants/year is far below the world average of 142.8 TKRs per 100,000 inhabitants/year,^8^ highlighting the significant deficit in healthcare.

TKR is a safe procedure. However, it is a major surgery aimed at elderly patients and is subject to possible complications.^10^ Most patients undergoing TKR have multiple comorbidities, which can significantly contribute to a higher number of complications.^9^ This surgery has acute risks associated with bleeding, anesthesia, cardiac and pulmonary complications, as well as thromboembolic phenomena, with complications occurring in 3% to 24% of cases.^11^ Some of these patients will require postoperative monitoring in intensive care units. In an effort to contain costs and optimize healthcare resources, it is important to identify patients who present significant risk factors for complications or require greater levels of postoperative surveillance following TKR.^12^ Similarly, other procedures historically treated as inherent to TKR may add cost and morbidity to the patient and currently need to be reviewed. A better definition of the need for postoperative surveillance in the ICU, the reservation and transfusion of blood products, and a reduction in the time to decision can optimize the allocation of finite resources in the healthcare system.

The main objective of the study was to validate the protocol for performing TKR without admission to the ICU in the postoperative period. The secondary objective was to evaluate the blood transfusion rate for the same specific group of patients after surgery.

## MATERIALS AND METHODS

After approval of the study by the institution's Research Ethics Committee, 270 cases of TKR performed between January 2020 and December 2021 were retrospectively evaluated.

The study was approved by the Research Ethics Committee of the Hospital Universitário Pedro Ernesto/UERJ (opinion number: 5.772.643).

All patients undergoing TKR during the study period had followed the screening protocol defined jointly by orthopedic surgeons, anesthesiologists, and clinicians after reviewing the literature. The seven criteria used for screening were: age up to 75 years, body mass index up to 35kg/m^2^, ASA (American Society of Anesthesiologists) *score* classification I or II, non-smoker, no previous history of ischemic disease (coronary or cerebral), creatinine clearance greater than 60 mL/min, hemoglobin greater than 12 g/dL, and osteoarthritis with deformity treatable with primary prosthesis (posterior stabilization – PS), which may be fixed or rotating. This protocol was published on protocols.io website.^13^ (Table 1)

**Table 1:** Criteria for performing TKR without ICU reservation.

Age ≤ 75 years
Body mass index ≤ 35kg/m2
ASA score* 1 or 2
Non-smoker
No history of ischemic disease (coronary or cerebral)
Creatinine clearance ≥ 60 mL/min
Hemoglobin ≥ 12 g/dL
Resolution with primary prosthesis

Source: Loures et al.^13^ ASA, American Society of Anesthesiologists; TKR, total knee replacement; ICU, intensive care unit.

Patients underwent a nursing assessment, during which they were identified, their height was measured, and their body weight was assessed, allowing for the calculation of their body mass index (BMI). Next, the orthopedic surgeon examined each patient, assessing the mechanical axis, range of motion, and degree of joint involvement (using the radiographic classification described by Ahlback and modified by Keyes et al.).^14,15^ The clinician confirmed that each patient met the defined parameters using a highly sensitive admission protocol. All patients included in the study underwent TKR without the use of a tourniquet. Thirty minutes before surgical access, intravenous infusion of tranexamic acid (Transamim®) was administered at a dose of 10 mg per kilogram of body weight, and antibiotic prophylaxis with 2 g of first-generation cephalosporin (Kefazol®) was administered. After surgery, patients were sent to the orthopedic ward, where they got IV pain meds and their pain was checked every four hours using a visual analog scale. On the morning of the first postoperative day, radiographic control was performed, which included anteroposterior and profile radiographs of the knee and leg, measurement and recording of the postoperative red blood cell count, and initiation of ambulation with the assistance of a physical therapist. Patients with insufficient data in their medical records were excluded.

Descriptive analysis was performed using measures of central tendency and dispersion for numerical data and frequency and percentage for categorical data. The inferential analysis was composed to compare numerical data between two moments of numerical data using the Student's t-test for paired samples; and for the interrelationships of variables using Pearson's correlation coefficient and Student's t-test for independent samples. The normality of data distribution was assessed using the Shapiro-Wilk test and graphical analysis of histograms. Corresponding nonparametric statistical tests were applied to reject the hypothesis of data normality. The criterion for determining significance was set at 5%. Statistical analysis was performed using SPSS version 26 software.

## RESULTS

The study included 270 patients who underwent TKR between January 2020 and December 2021, following the predefined screening protocol. The sample consisted of 182 female patients and 88 male patients. The average age was 65.5, ranging from 39 to 75 years. The average body mass index was 30.3kg/m^2^, ranging from 20.6 to 35kg/m^2^. The sample characteristics are shown in Table 2. Only one patient required ICU monitoring after surgery due to hemodynamic instability, giving the screening protocol a positive predictive value of 99.6%.

**Table 2:** Sample characterization.

Variable	n	Average	SD	median	IIQ	Minimum	Maximum
Clinic							
Age (years)	270	65.5	6.1	66	61-70	39	75
Weight (kg)	208	78.1	10.8	78.8	70.0-85.2	53	109
Height (m)	208	1.61	0.09	2	1.54-1.68	1.35	1.82
IMC (kg/m2)	217	30.3	3.6	30.8	28.0-33.3	20.6	35

IIQ, interquartile range.

The fixed base prosthesis was used in 176 patients and the removable base in 94 patients. The length of hospital stay did not follow a normal distribution (p < 0.0001), so the most appropriate measures for data analysis were the median and quartiles, with the mean presented for reference purposes only. There was no difference in length of hospital stay between age groups (p = 0.42), side operated (p = 0.058), ASA score (p = 0.36), and presence or absence of associated clinical comorbidities. Male patients had a significantly longer hospital stay (p = 0.015) than female patients, as did patients who used fixed-base prostheses compared to rotary-base prostheses (p = 0.002). Table 3 shows the relationship between length of hospital stay and categorical variables.

**Table 3:** Length of hospital stay in relation to categorical variables.

Variable	n	Length of hospital stay (days)			p-value
		Average	Median	IIQ	
**Age group**					
≤ 60 years old	56	4.8	4	4-6	0.42
61 to 65 years old	76	4.9	5	4-6	
66 to 70 years old	74	5.2	4	3-5.3	
> 70	64	4.9	5	4-6	
**Sex**					
Male	88	5.5	5	4-6	0.015
Women	182	4.7	4	3.8-6	
**Side**					
Right	144	4.7	4	3-6	0.058
Left	126	5.3	5	4-6	
**ASA**					
ASA 1	27	4.6	4	3-6	0.36
ASA 2	240	5.0	5	4-6	
**Diabetes**					
Yes	61	5.3	5	3.5-6	0.65
No	209	4.9	5	4-6	
**High blood pressure**					
Yes	206	4.9	5	4-6	0.85
No	64	5.1	4.5	4-6	
**Hypothyroidism**					
Yes	18	4.9	4.5	4-6	0.85
No	252	5.0	5	4-6	
**Obesity**					
Yes	130	4.7	5	4-6	0.092
No	87	5.4	5	4-6	
**Type of prosthesis**					
Fixed	176	5.2	5	4-6	0.002
Rotatory	94	4.5	4	3-5	

IIQ, interquartile range (Q1-Q3). Kruskal-Wallis ANOVA for comparison between four categories and Mann-Whitney test for two categories.

In the sample of 201 patients who followed the red blood cell collection and recording protocol on the morning of the first postoperative day, Student's t-test showed a significant variation (p < 0.0001) in hemoglobin from preoperative (13.4 ± 1.3 g/dL) to postoperative (11.0 ± 1.6 g/dL), with a mean decrease of 2.4 g/dL (± 1.3 g/dL), corresponding to 17.7% (± 9.2%). Table 4 shows the pre- and postoperative hemoglobin levels and their variation.

**Table 4:** Pre- and postoperative serum hemoglobin levels.

**Hemoglobin**	**Average**	**SD**	**Median**	**IIQ**	**Minimum**	**Maximum**
Hemoglobin - pre (g/dL)	13.4	1.3	13.4	12.5 to 14.3	10	16.4
Hemoglobin - pre (g/dL)	11	1.6	11.1	10 to 11.9	7.1	16
Hemoglobin - post (g/dL)	-2.4	1.3	-2.4	-3.3 to -1.6	-6.8	1.1
Hemoglobin - delta (g/dL)	-17.7	9.2	-17.7	-24.4 to -11.5	-46.6	7.4
Hemoglobin - delta (%)						

SD, standard deviation; IQR, interquartile range (Q1-Q3). Kruskal-Wallis ANOVA for comparison between four categories and Mann-Whitney test for two categories.

Preoperative hemoglobin levels were relatively higher in male patients (p < 0.0001), in the age group under 60 years compared to those over 70 years (p = 0.038), and in patients without systemic arterial hypertension (SAH) (p = 0.029). Although there was a significant decrease in serum levels, no patient presented hemodynamic symptoms or clinical indications for blood transfusion. Table 5 shows the variation in hemoglobin and its relationship with categorical variables.

**Table 5:** Variation in hemoglobin.

Variable	n	Hb pre (g/dL)		p-value	Δ Hb (%)		p value
Age group							
≤ 60 years old	39	13.7	1.4	0.038	−16.2	8.7	0.43
61 to 65 years old	58	13.5	1.1		−18.7	7.5	
66 to 70 years old	57	13.4	1.4		−18.6	10.8	
> 70	47	12.9	1.2		−16.8	9.2	
**Sex**							
Male	61	14.0	1.4	< 0.0001	−16.4	9.9	0.16
Women	140	13.1	1.1		−18.3	8.8	
**Side**							
Right	102	13.4	1.3	0.80	−17.8	9.6	0.90
Left	99	13.4	1.2		−17.7	8.7	
**ASA**							
ASA 1	18	13.6	1.4	0.56	−15.4	6.2	0.27
ASA 2	180	13.4	1.3		−17.9	9.4	
**Diabetes**							
Yes	45	13.4	1.3	0.83	−17.7	8.9	0.96
No	156	13.4	1.3		−17.7	9.2	
**High blood pressure**							
Yes	156	13.2	1.2	0.029	−18.1	9.2	0.24
No	45	13.8	1.3		−16.3	9.1	
Hypothyroidism							
Yes	13	13.2	1.4	0.55	−14.2	5.4	0.037
No	188	13.4	1.3		−18.0	9.3	
**Obesity**							
Yes	101	13.4	1.3	0.52	−18.2	8.4	0.49
No	63	13.3	1.4		−17.2	10.0	
**Type of prosthesis**							
Fixed	124	13.2	1.2	0.055	−17.7	8.9	0.98
Roundabout	77	13.6	1.3		−17.8	9.6	

ANOVA for one factor to compare four categories and *Student's* t-test for independent samples for two categories. According to Tukey's multiple comparison test, at a level of 5%, it was found that the ≤ 60 age group had significantly higher preoperative Hb levels than the > 70 age group.

## DISCUSSION

The proposed clinical screening protocol proved to be effective and safe, allowing for an increase in the number of centers qualified to perform TKR in the country and optimizing costs. Knee osteoarthritis is a common, limiting, and very symptomatic disease. Although it affects 250 million people worldwide, the vast majority of patients do not receive adequate treatment,^5^ which has an impact on productivity and the quality of life of patients. The Brazilian population includes 31.2 million people over the age of 60.^16^ Although increased life expectancy is a social achievement, only 15% of the elderly population has some form of health care plan, with the remaining 85% dependent exclusively on the Unified Health System (SUS). TKR is a surgical procedure capable of restoring function, relieving pain, and restoring autonomy to patients with treatment-resistant symptoms, but it is a costly procedure. In Brazil, 6.29 TKR/100,000 inhabitants/year are performed, far below the world average of 142.8 TKR/100,000 inhabitants/year, evidencing a significant deficit in care.^9^ The creation and validation of protocols that allow for the optimization of resources and expansion of safe treatment options for patients is essential, especially given the expected growth in the incidence of this disease. Ro et al.^17^ highlight that knee OA has a higher incidence in females, with a ratio of 2 to 3 women for every man. The sample in this study consisted of 182 female patients and 88 male patients, with a ratio of 2.06 women to each man, consistent with the literature.^18^

Most patients (79%) were between 60 and 75 years of age, consistent with the criteria for TKR. Although there is little evidence in the literature to define an age limit, the proposed protocol included patients up to 75 years of age in the sample. Kuperman et al.,^19^ in their meta-analysis, emphasized that age alone cannot be a contraindication for TKR, but the risk of clinical complications increases proportionally. Liodakis et al.^20^ found that age over 75 years was a predictor of complications and increased length of hospital stay, and Klausing et al.^11^ described age as an important factor linked to adverse events after TKR, with a 2% increase in risk for each year added to the age of 75 years. *An ASA score* greater than 2 or *creatinine clearance* less than 60 mL/min are, in isolation, significant risk factors for ICU admission after arthroplasty, so these patients were excluded until the risk factors were adjusted.^21^ Smoking is clearly linked to a higher number of complications after TKR, with a significant adverse effect and a higher rate of reoperation due to infection (RR 1.82),^22^ pneumonia, and surgical wound healing problems.^23^ Sahota et al.^24^ evaluated 1,251 smokers who underwent arthroplasty and found a significantly higher rate of clinical complications and hospital readmission, as well as a higher probability of surgical site infection, a catastrophic problem with high mortality. The relative risk for any adverse events after TKR in patients who smoke can be as high as 65.13 times.^25^ Therefore, patients should stop smoking at least six weeks before the procedure.

Obesity is clearly linked to the onset and progression of osteoarthritis,^3^ and is a major risk factor for orthopedic and clinical complications, especially in candidates for arthroplasty. The study protocol included patients with a BMI of up to 35kg/m^2^ (grade I obesity), who represented 59.9% of the sample. Patients with a BMI greater than 35kg/m^2^ were excluded because they had a higher chance of reoperation (p = 0.021), longer surgical time (p < 0.001), and longer hospital stay (p = 0.005).^26^ The group that underwent rotary TKR had a shorter hospital stay. Although there is no clear preference in the literature, this fact may be related to the greater severity of osteoarthritis in the fixed TKR group, since patients with bone defects and ligament instability on physical examination were referred to the fixed implant group.

The preoperative serum hemoglobin level is directly related to the complication rate, with each point below 12 g/dL increasing the chance of adverse events by 16%.^11^ Therapeutic changes in recent years have dramatically reduced blood loss and the need for transfusion during or after TKR. Huang et al.^27^ demonstrated that prophylactic use of tranexamic acid without tourniquet use resulted in lower hemoglobin loss, lower transfusion rates, and better postoperative clinical outcomes. Riggle et al.^28^ found no difference in postoperative red blood cell count between patients operated with and without the use of pneumatic tourniquets, although the operating time was longer in the first group. The protocol was applied to all patients in the study, where no blood transfusion was necessary. A total of 270 blood types were identified and 540 red blood cell concentrates were reserved, but no bags were used, which only increased the cost and complexity of the procedure. With the modernization of procedures and advances in surgical techniques, blood typing can be performed only when necessary, postoperatively, without compromising patient safety.^29,30^

Postoperative complications can occur in between 3% and 24% of patients and are the main source of healthcare system resource consumption.^9,11^ Especially in elective surgeries, the use of risk stratification protocols reduces the risk of adverse events, indicating possible complicating clinical parameters, optimizing results, and creating less complex arthroplasty centers in order to reduce waiting times and improve the use of resources. Several factors such as hemoglobin levels, BMI > 35kg/m^2^, and smoking can be corrected prior to the procedure, increasing patient safety and improving results. The study has some limitations. The screening protocol used has high sensitivity, achieving a positive predictive value of 99.6%, but has low specificity. Thus, the possible relaxation of some criteria may enable a greater number of patients to be treated in less complex centers. The pathologies were assessed by their presence, as dichotomous factors, and were not stratified by severity (with the exception of obesity). The study was unable to identify which patients will require intensive care in the postoperative period, but it clarified that, following the proposed protocol, the chance of adverse events is very small. Kamath et al.^21^ used a similar screening protocol, but reserved ICU beds when patients had two or more factors, a method that may guide a more flexible approach. The major advantage of the study was the validation of a simple and easily applicable screening protocol, allowing for the expansion of referral centers for arthroplasty, optimizing healthcare resources, and preserving patient safety.

## CONCLUSION

The proposed triage protocol proved to be effective and safe, allowing TKR to be performed without the need for prior ICU bed reservation, blood typing, and/or blood product reservation, without compromising patient safety.
